# Web-Based Therapist-Guided Mindfulness-Based Cognitive Behavioral Therapy for Body Dysmorphic Disorder: Pilot Randomized Controlled Trial

**DOI:** 10.2196/55283

**Published:** 2024-06-12

**Authors:** Camrie Kerry, Prabhdeep Mann, Nazanin Babaei, Joel Katz, Meysam Pirbaglou, Paul Ritvo

**Affiliations:** 1 School of Kinesiology and Health Sciences York University Toronto, ON Canada; 2 Department of Psychology University of Victoria Victoria, BC Canada; 3 Department of Psychology York University Toronto, ON Canada

**Keywords:** body dysmorphic disorder, BDD, dysmorphophobia, obsessive-compulsive and related disorders, OCD, internet-delivered cognitive behavior therapy, iCBT, cognitive behavior therapy, mindfulness-based cognitive therapy, mindfulness, eMental health, randomized controlled trial

## Abstract

**Background:**

Internet-based cognitive behavioral therapy (CBT) and stand-alone mindfulness meditation interventions are gaining empirical support for a wide variety of mental health conditions. In this study, we test the efficacy of web-based therapist-guided mindfulness-based cognitive behavioral therapy (CBT-M) for body dysmorphic disorder (BDD), a psychiatric disorder characterized by preoccupations with perceived defects in appearance.

**Objective:**

This study aims to determine whether CBT-M for BDD delivered on the web is feasible and acceptable and whether mindfulness meditation adds to CBT treatment effects for BDD.

**Methods:**

In this 8-week, 2-arm, parallel pilot randomized controlled trial, n=28 adults (aged between 18 and 55 years) were randomly allocated to an experimental group (web-based therapist-guided CBT-M) or a control group (web-based therapist-guided CBT). Study retention, accrual, and intervention adherence were assessed, along with self-report measures for BDD, depression, anxiety, and pain intensity taken at baseline and postintervention.

**Results:**

This study was feasible to implement and deemed acceptable by participants. After 8 weeks, significant improvements were found on all outcome measures for both treatment groups, and large between-group effect sizes favoring CBT-M were found for BDD symptom severity (*d*=–0.96), depression (*d*=–1.06), pain severity (*d*=–1.12), and pain interference (*d*=–1.28). However, linear mixed models demonstrated no significant differences between the groups over 8 weeks.

**Conclusions:**

The results suggest that mindfulness meditation may add to beneficial web-based CBT treatment effects for BDD. An adequately powered randomized control trial of web-based CBT-M is warranted.

**Trial Registration:**

ClinicalTrials.gov NCT05402475, http://clinicaltrials.gov/ct2/show/NCT05402475

## Introduction

### Background

Body dysmorphic disorder (BDD) is a psychiatric disorder marked by excessive preoccupations with perceived physical defects that are slight or imperceptible to others [1]. Individuals with BDD perform repetitive behaviors such as analyzing perceived defects in reflective surfaces, seeking reassurance from others, excessive grooming, and camouflaging with oversized clothing or makeup [2]. Preoccupations with appearance and associated behaviors often lead to significant distress and functional impairment, including difficulties attending work or school [3] that render many (nearly 30%) housebound [4,5]. Furthermore, suicidal ideation and suicide attempts are common in BDD-affected populations [6], with completed suicide rates nearly 45 times higher than the general population’s [7], reflecting the debilitating morbidity of BDD.

BDD symptoms typically arise during adolescence and impact 1.7% to 2.4% of the general population over the life span [6-9]. While population-based studies indicate a higher proportion of BDD-affected individuals are female (nearly 60%), demographics, body areas of concern, symptom severity, clinical features, and impairment appear similar between genders [2,8,9]. However, these and other BDD-related data may be approximate, as BDD is understudied, underdiagnosed, and undertreated [6], partly because individuals reluctantly disclose BDD symptoms due to feelings of shame [10]. The reluctance to disclose may lead BDD-affected individuals to seek out cosmetic enhancements at an observed prevalence of 6% to 15% [11-13].

Individuals affected by BDD typically do not benefit from surgical treatment, while cognitive behavioral therapy (CBT) offers an efficacious treatment alternative [14]. Mindfulness meditation has also been associated with reductions in body image distress through procedures that support appearance acceptance and present-moment awareness [15]. Despite the potential of multiple treatment options, several factors may impede access to BDD treatment, including poor insight, lower socioeconomic status, shame, geographical barriers, and psychotherapeutic ambivalence [16-18].

Surveys have identified that only 17.4% of individuals who actively sought psychotherapy services for BDD received CBT, despite its empirical support [5,17]. Internet-based CBT for BDD may increase treatment access, with research investigating the efficacy of internet-based CBT (iCBT) for BDD identified as encouraging. In Sweden, Enander et al [19] conducted a 12-week randomized controlled trial (RCT) comparing iCBT for BDD with web-based supportive therapy. Results revealed a between-group effect size of *d*=0.95 (95% CI 0.52 to 1.38) after treatment, indicating substantially reduced BDD outcomes for the intervention group when compared to supportive therapy. In 2020, Wilhelm et al [20] developed and pilot-tested a CBT digital service, marking the first smartphone-delivered individual CBT treatment for BDD. Although substantial symptom reduction was reported after the 12-week open trial (*d*=2.60), the efficacy indications must be interpreted cautiously as this study was an underpowered, nonrandomized trial (n=10 participants) in which depression outcomes did not meaningfully reduce. Ultimately, these aforementioned factors underscore the importance of expanding BDD research to identify appropriate and accessible clinical interventions.

While iCBT interventions address multiple BDD treatment barriers and demonstrate impressive empirical support, there are readily identified needs for improvement. First, CBT interventions for BDD appear to minimally reduce comorbid depression [14] which is unfortunate given that individuals with BDD have high rates of suicidal ideation, functional impairment, and major depressive disorder [2,3,6]. There is evidence from existing web-based therapist-guided RCTs that mindfulness-based CBT (CBT-M) interventions reduce depression symptoms [21,22]. Although these trials did not include BDD participants, they demonstrate that accessible and effective treatments exist for depressed subpopulations. While previous web-based CBT for BDD protocols have incorporated mindfulness [23], CBT-M interventions deliberately emphasize mindfulness practice linked to techniques for engaging in self-compassion [24], self-acceptance, and nonjudgement [25], demonstrating significant promise for reducing body image distress [15]. This is essential to consider for those affected by BDD, given their vulnerability to negative self-appearance fixations. Findings from a study focused on the short-term effectiveness of targeting intrusive appearance-linked thoughts through mindfulness meditation identified that positive affect increased in BDD individuals compared to healthy controls [15]. As mindfulness techniques implicitly and explicitly emphasize relaxation, nonjudgement, and nonreactivity [26], participants may develop a strengthened propensity to engage in CBT practices by identifying maladaptive beliefs that drive BDD and are found to be modifiable.

### Aims of the Study

While the efficacy of web-based therapist-guided CBT-M for BDD is heretofore unknown, reductions in BDD symptoms already achieved in web-based CBT and mindfulness meditation interventions suggest a combined treatment approach is an important investigative priority. Furthermore, the current trial isolates mindfulness components to better understand their treatment effects.

Curiously, despite self-injurious behavior patterns such as skin-picking, excess exercise, suicide attempts [2] and cosmetic surgery engagement [27], research investigating physical pain in BDD appears sparse. In extreme cases, self-mutilation, self-surgery [2] and requests for healthy limb amputations [28] have been reported in individuals affected by BDD. This notable gap in pain investigation was additionally prioritized in this study.

We anticipate that web-based intervention implementation will be feasible, indicated by rates of accrual, retention, and attendance at therapist-guided calls, and acceptability will be demonstrated by responses on the NexJ Program Experience survey. Furthermore, we hypothesize that the CBT-M group participants will reveal greater posttreatment reductions in BDD, depression, anxiety, and pain according to between-group effect sizes, and preliminary efficacy for both groups will be demonstrated by quantitative outcomes (symptom reductions) assessed at baseline and postintervention.

## Methods

### Participants

Recruitment was undertaken through advertisements on web-based platforms (Facebook [Meta Platforms, Inc], Reddit [Reddit, Inc], and CloudResearch [Prime Research Solutions LLC]), and direct referrals were accepted from the Centre for Addiction and Mental Health (Toronto, Ontario).

### Inclusion Criteria

US or Canadian residents aged between 18 and 55 years; fluent in English; having smartphone access; BDD diagnosis based on Body Dysmorphic Disorder Questionnaire (BDDQ) responses [29] must answer “yes” to the following questions: (1) Are you worried about how you look? (2) Do you think about your appearance problems a lot and wish you could think about them less? Must answer “yes” to any of the following questions: (1) How has this problem with how you look affected your life? Has it often upset you a lot? (2) Has it often gotten in the way of doing things with friends, dating, your relationships with people, or your social activities? (3) Has it caused you any problems with school, work, or other activities? (4) Are there things you avoid because of how you look? Eligible participants must additionally indicate that they spend ≥1 hour each day thinking about how they look.

### Exclusion Criteria

Individuals whose self-reports are congruent with the *DSM-5* (*Diagnostic and Statistical Manual of Mental Disorders* [Fifth Edition]) criteria for: eating disorder, bipolar disorder, borderline personality disorder, schizophrenia (or other primary psychotic disorder) or severe substance abuse disorder or addiction; disclosure of imminent intent or attempted suicide in the past 6 months; concurrent psychological treatment; and no smartphone access.

### Sample Size

Following Julious’ [30] pilot recommendations, 24 participants were allocated to 2 comparison groups, and 28 participants were enrolled for a final recruitment target of 24, assuming 15% (4/28) attrition.

### Screening

Participants were screened on the web with Survey Monkey (Survey Monkey, Inc), a HIPAA (Health Insurance Portability and Accountability Act)-compliant platform for safeguarding data collection. The BDDQ [29] was used to detect BDD in combination with a psychological history interview.

### Data Collection

Participants completed a web-based consent form and 4 questionnaires: the Body Dysmorphic Disorder-Symptom Scale [31], the Patient Health Questionnaire-9 (PHQ-9) [32], the Generalized Anxiety Disorder-7 Scale (GAD-7) [33], and the Brief Pain Inventory (BPI) [34]. The web-based assessment used a SurveyMonkey link with the additional safeguard of a unique study ID number.

Upon questionnaire completion, participants provided phone numbers for receipt of therapist-guided calls associated with the NexJ Connected Wellness platform. Self-reported psychometric data were collected from participants at baseline (T1) and postintervention (T2). Participants were compensated US $30 for intervention participation and measurement completion.

### Randomization

A 1:1 ratio randomization schedule was used with randomly selected block sizes of 4 and 2 treatment arm allocations (CBT-M or CBT) based on a randomization sequence generator. Participants were allocated to treatment groups after providing consent and before baseline measurement.

### Interventions

After participants were randomly allocated to treatment, the principal investigator (Dr Paul Ritvo) assigned participants to student-therapists for weekly guided calls. Participants were additionally connected to the NexJ Connected Wellness platform to access BDD-related content and to enable secure message exchanges with their therapist.

CBT-M participants received 24/7 accessible CBT and mindfulness content through NexJ Connected Wellness. The content was based on 2 previous internet-based CBT-M RCTs with distressed students and individuals diagnosed with major depressive disorder [21,35]. Altogether, 8 workbook chapters focused on (1) perfectionism, unchangeable physical features, media influences and comparisons; (2) automatic negative thoughts, repetitive behaviors, and mindful acceptance; (3) mindful nonreactivity, cognitive restructuring, and behavior modification; (4) body-based assumptions and befriending your body; (5) overcoming avoidance, and internal versus external body image; (6) diaphragmatic breathing, loving-kindness meditation, and cultivating compassion to others and self; (7) bringing health from most preferred body parts to least preferred body parts, shifting to a neutral focus, and adjusting assumptions; and (8) objective self-talk, shifting attentional focus, and continuing the intervention independently.

While the CBT-only group did not receive mindfulness meditation content (mindful acceptance, mindful nonreactivity, loving-kindness meditation, compassion to others and self, and shifting attentional focus), both groups were provided with 8 modules and engaged in 1 hour of self-directed CBT-M or CBT content per week. The CBT-M group was provided with ~135 minutes of mindfulness audio to select from.

Client-centered therapist calls were conducted for 60 minutes per week over 8 weeks, which involved discussing module content and progress. All study therapists attended group training and 1-on-1 supervision sessions weekly.

### Measures

The primary feasibility outcomes were measured by an accrual rate (total number of enrolled participants divided by the number of months recruitment occurred), retention (percentage of participants who completed the 8-week intervention from randomization to completion of postintervention measures) and adherence (percentage of participants attending weekly and scheduled therapist-guided calls). The primary acceptability outcome was measured by the NexJ Program Experience survey, a 7-item questionnaire (1-5 rating scale) developed by NexJ Health Inc wherein higher scores represent greater participant satisfaction. The Body Dysmorphic Disorder Symptom Scale (BDD-SS), a reliable and valid self-report questionnaire was used to examine the severity of BDD symptoms [31]. This study uses the 7 severity items, with higher scores representing greater severity (of symptoms), up to a sum score of 70.

Secondary outcomes for depression, anxiety and pain were measured using the PHQ-9 [32], the GAD-7 [33], and the BPI Short Form [34], respectively. The PHQ-9 is a reliable and valid 9-item self-report questionnaire for depression screening and severity, with a sensitivity of 88% and specificity of 88% [32]. The GAD-7 is a valid measure for screening and assessing the severity of generalized anxiety disorder, with a sensitivity of 89% and a specificity of 82%, indicating good reliability [33]. Lastly, the BPI (Short Form) is a reliable self-report questionnaire for physical pain experiences, using 4 pain severity items and 7 pain interference items. The BPI has demonstrated good to excellent validity and reliability [34].

### Statistical Analyses

SPSS Statistics (version 28.0; IBM Corp) for Windows was used to analyze self-reported data. Numeric variables were presented as means and SDs, and categorical data were presented as frequencies and percentages.

Independent sample 2-tailed *t* tests and the Fisher exact test for all numeric and categorical variables were used to detect differences between study groups at baseline and between study completers and dropouts to determine whether missing postintervention measurements were considered missing at random. Linear mixed model (LMM) analyses for repeated measures were used in an intention-to-treat approach. An unstructured restricted maximum likelihood approach was used for the main analysis (“Results” section). Fixed effects, including group and time, and their interaction (group × time) were evaluated. Cohen *d* within-group and between-group effect sizes were evaluated with means, SDs, and correlations calculated for all participants with completed data (n=18).

Pearson *r* bivariate correlations were computed to assess the relationship between BDD-SS and BPI scores.

### Ethical Considerations

This 8-week 2-arm pilot RCT used a web-based software platform, NexJ Connected Wellness (NexJ Health Inc) and was approved by York University Research and Ethics and registered with ClinicalTrials.gov (NCT05402475).

## Results

### Participant Flow

**Figure 1:**
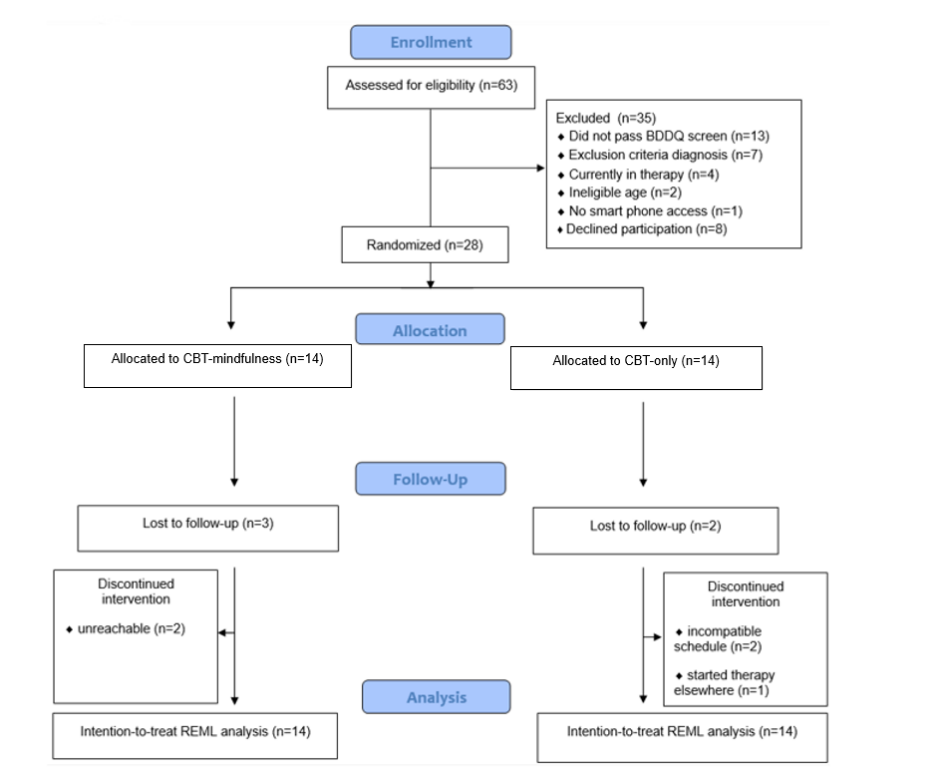
CONSORT (Consolidated Standards of Reporting Trials) participant flow diagram. BDDQ: Body Dysmorphic Disorder Questionnaire; CBT: cognitive behavioral therapy; REML: restricted maximum likelihood.

The CONSORT flow diagram (Figure 1) illustrates the recruitment and flow of 63 adults (aged between 18 and 55 years) who were screened and interviewed to determine eligibility (September 2022 to February 2023). Of those interviewed, 35 adults were ineligible because: they did not pass BDDQ screening (n=13), were excluded due to psychiatric diagnosis (n=7), current therapy engagement (n=4), ineligible age (n=2), lack of smart phone access (n=1), or decided not to participate (n=8). A total of 28 adults met inclusion criteria and provided informed consent to participate. Demographic and psychological characteristics for both CBT-M and CBT groups are presented in Table 1.

**Table 1:** Demographic and psychological characteristics for both groups at baseline (N=28).

Variable			CBT-M^a^		CBT^b^		*P* value	
Age (years), mean (SD)			31.14 (7.81)		34.43 (11.33)		.93	
**Gender, n (%)**								.38
	Female	7 (50)		10 (71.4)				
	Male	5 (35.7)		4 (28.6)				
	Other	2 (14.3)		0 (0)				
**Ethnicity, n (%)**								.53
	White	8 (57.1)		7 (50)				
	Black	2 (14.3)		3 (21.4)				
	South Asian	2 (14.3)		1 (7.1)				
	East Asian	0 (0)		2 (14.3)				
	Latin American	0 (0)		1 (7.1)				
	Multiethnic	2 (14.3)		0 (0)				
**Highest level of education, n (%)**								.58
	High school	3 (21.4)		1 (7.1)				
	College	1 (7.1)		3 (21.4)				
	Bachelor’s degree	8 (57.1)		6 (42.9)				
	Master’s degree	2 (14.3)		3 (21.4)				
	Other	0 (0)		1 (7.1)				
**Marital status, n (%)**								.70
	Married or common-law	7 (50)		5 (35.7)				
	Single	7 (50)		9 (64.3)				
**Psychological variables, mean (SD)**								
	BDD-SS^c^ (Severity)	35.50 (10.47)		41.64 (11.24)		.15		
	PHQ-9^d^	10.07 (3.69)		11.36 (5.44)		.47		
	GAD-7^e^	8.36 (4.34)		8.57 (4.13)		.90		
	BPI^f^ (severity 0-10)	2.57 (2.2)		2.66 (2.29)		.92		
	BPI (interference 0-10)	2.26 (2.32)		3.41 (3.27)		.29		

^a^CBT-M: mindfulness-based CBT.

^b^CBT: cognitive behavioral therapy.

^c^BDD-SS: Body Dysmorphic Disorder Symptom Scale.

^d^PHQ-9: Patient Health Questionnaire-9.

^e^GAD-7: Generalized Anxiety Disorder-7.

^f^BPI: Brief Pain Inventory.

### Intervention Feasibility

A total of 28 eligible participants enrolled during the 5 months of recruitment (September 2022-February 2023) for an accrual rate of 5.6 participants per month. A total of 10 enrollees did not complete the 8-week intervention for a 64% (18/28) retention rate.

All 18 retained participants completed 8 scheduled counseling calls, although 5 participants had to reschedule 1 call (n=5-CBT) and another 5 participants had to reschedule 2 calls (n=2-CBT-M and n=3-CBT). About 44% (8/18) of participants maintained attendance for scheduled weekly calls throughout the study.

### Intervention Acceptability

All 18 participants who completed the intervention provided program satisfaction ratings at 8 weeks. Participants rated their experience from 1-5 on the NexJ Program Experience survey (Table 2). The mean score for the question “How would you rate your overall program experience?” was 4.67 (SD 0.59), where 1 indicated “poor” and 5 indicated “excellent.” In total, 72% (13/18) of participants identified their overall experience in the study was “excellent” while 22% (4/18) of participants indicated their experience was “very good,” and one participant (0.06%; 1/18) indicated their experience was “good.” Other acceptance indications are found in Table 2.

**Table 2:** Results from the NexJ Program Experience survey postintervention. Satisfaction scores ranged from 1-5.

Satisfaction metric	CBT-M^a^, mean (SD)	CBT^b^, mean (SD)	Mean (SD)
How would you rate your overall program experience?	4.78 (0.67)	4.56 (0.53)	4.67 (0.59)
On average, how much time did you spend on the program each week?	3.89 (0.78)	3.78 (0.83)	3.83 (0.79)
To what extent did the program meet your needs?	4.56 (1.01)	4.44 (0.88)	4.5 (0.92)
How would you rate the ease of using our platform?	3.89 (1.27)	4.67 (0.50)	4.28 (1.02)
Please indicate your agreement with the following statement: The information in the modules helped me work towards my mental health goals	3.33 (1.23)	3.22 (1.20)	3.28 (1.18)
Overall, my experience with my therapist was:	5 (0)	4.78 (0.67)	4.89 (0.47)
How likely are you to continue using our platform over the next 6 months?	3 (1.5)	3.67 (1.12)	3.33 (1.33)

^a^CBT-M: mindfulness-based cognitive behavioral therapy.

^b^CBT: cognitive behavioral therapy.

### Preliminary Efficacy

#### Overview

As shown in Table 1, independent sample *t* tests and Fisher exact tests revealed no significant differences between groups on baseline measures for all assessed variables. This suggests that randomization allocation resulted in reasonably equivalent treatment groups. In addition, the main analysis did not include covariates within the statistical model. Independent sample *t* tests and Fisher exact tests revealed no significant differences at baseline in dropout patterns (those who stayed in vs dropped out): age (*P*=.41), gender (*P*=.84), education (*P*=.89), ethnicity (*P*=.25), marital status (*P*=.69), BDD-SS score (*P*=.82), PHQ-9 score (*P*=.55), GAD-7 score (*P*=.67), BPI (severity) score (*P=*.95), or BPI (interference) score (*P*=.84). As a result, missing postintervention measurements (n=10) were considered missing at random. Unstructured restricted maximum likelihood data (Table 3) and Cohen *d* effect sizes for each psychometric outcome, along with correlation coefficients between BDD and pain, are discussed below.

**Table 3:** Results from linear mixed model (LMM) analysis for changes in outcomes from baseline to postintervention between and within intervention groups (intention-to-treat using restricted maximum likelihood).

Outcomes		CBT-M^a^	CBT^b^	*d* (between-groups)		Group					Time					Group*×t*ime			
						*F* test (*df*)		*P* value		*F* test (*df*)			*P* value		*F* test (*df*)			*P* value	
**BDD-SS^c^ (Severity)**							5.12 (1, 25.63)		.03			76.93 (1, 22.24)		<.001			0.24 (1, 22.24)		.63
	Baseline	36.44 (12.39)	41.44 (12.2)		—^d^														
	8-weeks	11.44 (7.84)	19.89 (10.61)		–0.96														
	*d* (within-groups)	–2.41	–1.87		—														
**PHQ-9^e^**							2.52 (1, 26.42)		.13			32.8 (1, 24.26)		<.001			0.72 (1, 24.26)		.40
	Baseline	10.22 (4.3)	12 (5.79)		—														
	8-weeks	4.44 (2.3)	7.33 (3.39)		–1.06														
	*d* (within-groups)	–1.61	–0.91		—														
**GAD-7^f^**							0.001 (1, 23.64)		.98			7.11 (1, 19.53)		.02			0.05 (1, 19.53)		.83
	Baseline	8.89 (4.54)	8.56 (4.59)	—															
	8-weeks	5.67 (5.83)	5.22 (2.64)	–0.11															
	*d* (within-groups)	–0.61	–0.87	—															
**BPI^g^ (Severity)**							1.76 (1, 25.26)		.20			7.80 (1, 24.18)		.01			1.87 (1, 24.18)		.18
	Baseline	2.57 (2.2)	2.66 (2.29)	—															
	8-weeks	0.53 (0.76)	1.97 (1.77)	–1.12															
	*d* (within-groups)	–1.20	–0.33	—															
**BPI (Interference)**							3.63 (1, 25.95)		.07			15.04 (1, 25.42)		.001			0.08 (1, 25.42)		.78
	Baseline	2.26 (2.32)	3.41 (3.27)	—															
	8-weeks	0.16 (0.29)	1.60 (1.66)	–1.28															
	*d* (within-groups)	–1.34	–0.62	—															

^a^CBT-M: mindfulness-based cognitive behavioral therapy.

^b^CBT: cognitive behavioral therapy.

^c^BDD-SS: Body Dysmorphic Disorder Symptom Scale.

^d^Not available.

^e^PHQ-9: Patient Health Questionnaire-9.

^f^GAD-7: Generalized Anxiety Disorder-7.

^g^BPI: Brief Pain Inventory.

#### BDD-SS

**Figure 2:**
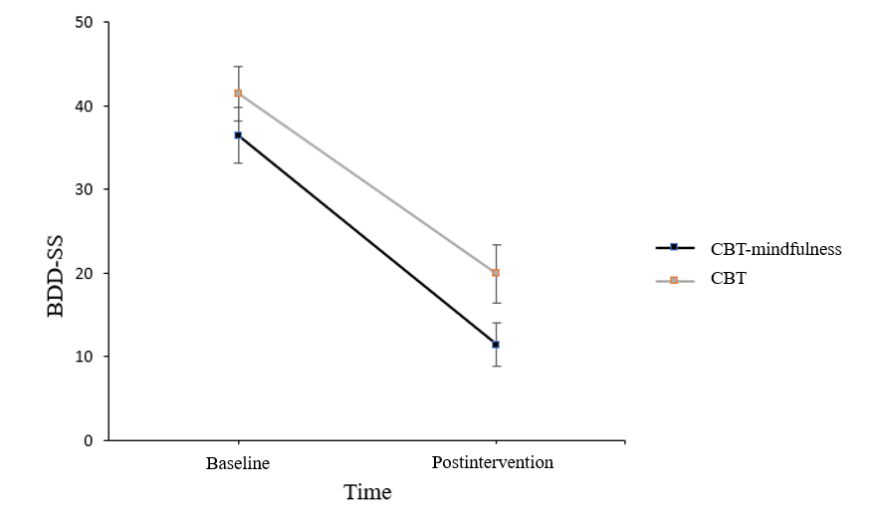
Body Dysmorphic Disorder Symptom Scale (BDD-SS) change from baseline to postintervention. Error bars represent standard error. CBT: cognitive behavioral therapy.

LMM analysis revealed a statistically significant main effect for group (*F*_1,25.63_=5.12, *P*=.03); a statistically significant main effect for time (*F*_1,22.24_=76.93, *P*<.001); but no significant group by time interaction (*F*_1,22.24_=0.24, *P*=.63). This suggests no statistically significant difference in BDD-SS change scores between the CBT-M and CBT groups from baseline to postintervention (Figure 2). A between- and within-groups evaluation of Cohen *d* effect sizes indicated a between-group effect size at 8 weeks of *d*=–0.96, and within-group effect sizes of *d*=–2.41 for CBT-M and *d*=–1.87 for CBT.

#### PHQ-9

Results from LMM analysis revealed a nonsignificant group by time interaction (*F*_1,24.26_=0.72, *P*=.40). In addition, no statistically significant main effects were found for group (*F*_1,26.42_=2.52, *P*=.13), although main effects for time did reveal statistical significance (*F*_1,24.26_=32.79, *P*<.001). Cohen *d* effect sizes revealed a between-group effect size of *d*=–1.06 at 8 weeks. The within-group effect size for CBT-M was *d*=–1.61 and *d*=–0.91 for CBT.

#### GAD-7

A statistically significant main effect for time (*F*_1,19.53_=7.11, *P*=.02) was found in the LMM analysis for GAD-7 scores. However, the main effects for group (*F*_1,23.64_=0.001, *P*=.98), and the group by time interaction (*F*_1,19.53_=0.05, *P*=.83) were not statistically significant. The Cohen *d* between-group effect size at 8 weeks was *d*=–0.11. The within-group effect size for CBT-M was *d*=–0.61 and *d*=–0.87 for CBT.

#### BPI (Severity)

LMM analysis revealed a statistically significant main effect for time (*F*_1,24.18_=7.80, *P*=.01) for BPI (severity). The main effects for group (*F*_1,25.26_=1.76, *P*=.20) and the group by time interaction (*F*_1,24.18_=1.87, *P*=.18) were not statistically significant. Cohen *d* effect sizes were calculated for between-group and within-group changes, revealing a between-group effect size at 8 weeks of *d*=–1.12, and within-group effect sizes of *d*=–1.20 for CBT-M and *d*=–0.33 for CBT.

A positive correlation between BDD-SS and BPI (severity) at baseline, *r=*0.29, was not statistically significant (*P*=.14).

#### BPI (Interference)

LMM analysis revealed a nonstatistically significant main effect for group (*F*_1,25.95_=3.63, *P*=.07), and group by time interaction (*F*_1,25.42_=0.08, *P*=.78) for pain interference; however, the main effect for time (*F*_1,25.42_=15.04, *P*=.001) was statistically significant. Cohen *d* evaluation of effect sizes revealed a between-group effect size of *d*=–1.28 at 8 weeks and within-group effect sizes of *d*=–1.34 for CBT-M and *d*=–0.62 for CBT.

A statistically significant, positive correlation was found between BDD-SS and BPI (interference) at baseline (*r=*0.56, *P*=.002).

## Discussion

### Overview

In this pilot RCT, the feasibility, acceptability, and relative efficacy of a web-based therapist-guided CBT-M intervention were compared with a web-based therapist-guided CBT-only intervention. Data indicate that, altogether, 28 participants were enrolled within 5 months of recruitment (September 2022 to February 2023) despite the inclinations of BDD-affected individuals to seek nonpsychiatric treatment at high rates [27]. These data challenge past data suggesting that individuals with BDD may be difficult to recruit for psychological treatment studies and support advocacy for more intervention access [14]. Adherence to scheduled phone-based counseling calls was another positive indicator, as 100% of study completers attended all 8 scheduled calls.

A moderate retention rate of 64% (18/28) suggests the RCT was implementable on the web with room for improvement. Effective implementation of the RCT was further indicated by satisfaction responses on the NexJ Program Experience survey. Most participants (13/18, 72%) indicated their program experience was “excellent,” and a good experience (or better) was reported by all study completers. Most participants (13/18, 72%) felt as though “almost all needs” were met, and participants found the NexJ Connected Wellness platform easy to use with a mean score of 4.28 (SD 1.02) out of an optimal score of 5.

Satisfaction ratings on module content were mixed, with 44% (8/18) indicating a neutral response, with 39% (7/18) finding modules helpful, and 17% (3/18) finding them not helpful. Overall discrepancies between satisfaction and low module ratings have been attributed to participant distress and the inconvenience of homework assignments in the CBT framework [36]. Findings also indicate a linkage between ambivalence about treatment tasks [37] and homework noncompliance, a challenging issue in CBT delivery [38]. Beyond the data presented above, additional data pertaining to participant use of module content were not obtained. However, 94% (17/18) of participants reported an excellent experience with their therapist during counseling calls, and only one indicated a “good” experience. These data align with other web-based CBT trials, amplifying the benefits of therapist support [19]. Of course, the use of an interactive platform with counseling calls makes it difficult to tease apart the benefits of web-based module access from the benefits of counseling calls.

Although no significant between-group differences from baseline to postintervention were observed on the BDD-SS, a statistically significant reduction in symptom severity from baseline to postintervention across both groups was found, as demonstrated by the LMM analysis. This supports our hypothesis that web-based therapist-guided CBT-M treatment for BDD will demonstrate preliminary efficacy.

Given the pilot sample, emphasis was placed on the between-group effect sizes for the proposed hypothesis that web-based therapist-guided CBT-M would be more effective than CBT alone. A between-group effect size of *d=–*0.96 at postintervention revealed that mindfulness meditation may contribute to beneficial treatment outcomes for BDD. Furthermore, very large within-group effect sizes were found for both CBT-M (*d*=*–*2.41), and CBT (*d*=*–*1.87) participants. These findings parallel results from a smartphone-based CBT for BDD RCT, where a within-group effect size of *d*=*–*2.26 (95% CI *–*2.93 to *–*1.58) was observed at 12 weeks [23]. This study achieved similar reductions in BDD after 8 weeks compared to Wilhelm et al’s 12-week intervention [23]. In combination with a web-based approach, a shorter intervention may prove cost-effective while reducing waitlists and clinician or therapist time [39]. These findings are relevant to the dearth of trained BDD clinicians and limited access to treatment [40].

Additional scales indicated comorbid symptom reductions. Accordingly, the large within-group effect sizes for web-based therapist-guided CBT (*d*=*–*0.91) and web-based therapist-guided CBT-M (*d*=*–*1.61), along with the statistically significant time effect, support the hypothesis that web-based therapist-guided CBT-M would reduce depression symptoms. Although large effect sizes were observed in both groups, web-based therapist-guided CBT-M was associated with larger effects. A large between-group effect size (*d*=*–*1.06) was also found, suggesting that mindfulness meditation in combination with CBT contributes to reductions in BDD-related depression. The effect size observed exceeds findings from 5 previous CBT for BDD RCTs reported in a previous meta-analysis [14], which revealed a moderate overall effect size (*d*=*–*0.49, 95% CI −0.76 to −0.22) for depression symptom reductions. Given that BDD and MDD are highly comorbid and have longitudinal associations [41], web-based therapist-guided CBT-M’s preliminary effectiveness for BDD-related depression is encouraging.

LMM analysis and Cohen’s *d* between-group effect size (*d*=*–*0.11) for anxiety revealed that treatment effects did not statistically differ between groups, rejecting our hypothesis that web-based therapist-guided CBT-M would be more effective than CBT alone. Although significant symptom severity improvements were observed after 8 weeks on GAD-7 scores in both groups, only a moderate CBT-M within-group effect size was found (*d=–*0.61). These findings are comparable to GAD-7 within-group effect sizes observed in a 16-week CBT for BDD RCT, *d*=0.65 [42].

With limited insight into whether BDD and pain are associated, the BPI was used to investigate variations in pain interference and severity. As an increased risk of self-injurious behavior is prevalent (eg, excessive exercise, restrictive eating, skin-picking, cosmetic surgery, self-surgery, suicide attempts, alcohol or drug dependency, and steroid abuse) [2,43], Pearson *r* correlation coefficients were calculated to assess the relationship between BDD and pain. While a statistically significant, positive correlation was found between BDD and pain interference, a weaker positive correlation was found between BDD and pain severity. Although pain exploration is a notable gap in BDD research, there is some evidential support for associations between body dissatisfaction and pain in eating disorder populations. Specifically, researchers found that body dissatisfaction may induce a greater sensitivity to bodily pain [44]. As body dissatisfaction is a cornerstone for BDD diagnosis, this research supports the preliminary insights in our study. Furthermore, in another study, when healthy individuals were confronted with distorted images of their own bodies, pain perception increased [45].

These data suggest that web-based therapist-guided CBT-M for BDD can reduce pain interference and pain severity scores, as demonstrated by statistically significant time effects. Although LMM did not reveal significant between-group differences, large between-group effect sizes for pain interference (*d*=*–*1.28), and pain severity (*d*=*–*1.12) were observed. The within-group effect sizes for pain severity greatly differed between treatment groups, with a large effect (*d*=*–*1.20) for web-based therapist-guided CBT-M and a small effect (*d*=*–*0.33) for CBT alone. Moreover, within-group effect sizes notably differed for pain interference (CBT-M *d*=*–*1.34 and CBT *d*=*–*0.62). The large effect sizes observed in the web-based therapist-guided CBT-M group for both pain severity and interference may be due to better chronic pain management obtained through mindfulness meditation practice [46].

### Limitations

Several limitations must be considered when interpreting this study’s findings. Given the pilot nature of this RCT, the small sample size limited the capacities to test for significance between treatment groups. In addition, the BDDQ screening tool was used for inclusion rather than a clinician-administered diagnostic measure. This may have resulted in an unrepresentative sample.

Although CBT-M module content and therapist-guided calls emphasized mindfulness meditation, data pertaining to participants’ historical meditation practices and active practice time throughout the 8-week intervention were not gathered, which limits understandings of the dose-response relationship [47]. Moreover, future CBT-M studies would optimally include a mindfulness measure such as the Five Facet Mindfulness Questionnaire [48] to establish linkage between potential mechanisms and BDD reductions.

### Conclusion

In this pilot RCT, two 8-week web-based interventions were compared in the treatment of BDD. Both interventions used therapist-guided CBT, but only one intervention combined CBT with mindfulness meditation approaches. Given the high accrual and adherence rates, moderate retention rate, and overall participant program satisfaction, this RCT was acceptable and feasible to implement. Preliminary efficacy was demonstrated for both active treatment groups, with suggestions that mindfulness meditation could add to CBT treatment effects for BDD and comorbid symptoms. In addition, a relationship between BDD and pain may be present, which requires additional investigation. Given that individuals with BDD may be housebound and have high rates of suicidal ideation and depression, prompt access to effective treatment is imperative. This pilot trial provides promising insight into BDD and short-term, web-based–accessible treatment for individuals with BDD.

## Appendix

Multimedia Appendix 1 CONSORT-eHealth checklist.

## Abbreviations

BDD: body dysmorphic disorder
BDDQ: Body Dysmorphic Disorder Questionnaire
BDD-SS: Body Dysmorphic Disorder Symptom Scale
BPI: Brief Pain Inventory
CBT: cognitive behavioral therapy
CBT-M: mindfulness-based cognitive behavioral therapy
DSM-5: Diagnostic and Statistical Manual of Mental Disorders (Fifth Edition)
GAD-7: Generalized Anxiety Disorder-7 Scale
HIPAA: Health Insurance Portability and Accountability Act
iCBT: internet-based cognitive behavioral therapy
LMM: linear mixed model
PHQ-9: Patient Health Questionnaire-9
RCT: randomized controlled trial
